# Effects of Tuna By-Product Meal on Growth, Whole-Body Mercury, Phosphorus Load, and Plasma Chemistry in Juvenile Greater Amberjack *Seriola dumerili*

**DOI:** 10.3390/ani14243711

**Published:** 2024-12-23

**Authors:** Amal Biswas, Shota Shirakawa, Satoshi Okimura, Tomoki Honryo, Hideki Tanaka

**Affiliations:** 1 Aquaculture Research Institute, Kindai University, Uragami 649-5145, Wakayama, Japan; 2 RegenWorks Co., Ltd., Iwayado, Odaka, Minamisoma 979-2157, Fukushima, Japan

**Keywords:** greater amberjack, tuna by-product meal, fish meal replacement, growth, Hg content, phosphorus load

## Abstract

The suitability of tuna by-product meal (TBM) as a replacement for fish meal (FM) in the diet of juvenile greater amberjack *Seriola dumerili* was investigated for its effect on growth, mercury (Hg) levels in the fish body, phosphorus (P) load, and plasma chemistry. FM was the main protein source in the control diet (C), and it was replaced by TBM at 25%, 50%, 75%, and 100% to formulate diets TM25, TM50, TM75, and TM100, respectively. The experiment was performed in triplicate, stocking 30 juveniles (mean weight: approximately 6.7 g) in each 500 L tank. After a 42 d rearing period, there were no significant differences in growth, nutrient digestibility, or productive value between diets C and TM25. However, a quadratic polynomial regression analysis suggested that the optimal dietary level of TBM against final weight was 14.5%. A significant linear correlation was observed between Hg in the diet and fish whole body, but the level was far lower, posing health risks to humans. Owing to the lower P intake of fish fed with the TBM-based diets, their P load was significantly reduced compared to the control group. Plasma levels of total protein, total cholesterol, and albumin in the TM75 and TM100 groups were significantly lower than those in the C, TM25, and TM50 groups. These results suggest that 14.5% of FM protein can be replaced by TBM protein in the diet of juvenile greater amberjack and that a significant reduction in P load can be achieved with a TBM-based diet.

## Simple Summary

In recent decades, there has been a global issue in searching for fish meal (FM) alternatives as a protein source due to the decreasing supply of FM, which caused a price surge. Although there is a scarcity of protein sources all over the world, a lot of by-products, despite high nutritive values, are wasted away, or the suitability of those by-products as protein sources has not been investigated in all commercially important species worldwide. Although tuna by-product meal (TBM) is considered one of the promising alternative protein sources, there is no information on its application as a protein source in the diet of juvenile greater amberjack *Seriola dumerili*. After replacing different levels of FM protein with TBM in the diet of juvenile greater amberjack, this study found that TBM can replace at least 14.5% of FM protein without compromising the growth and health status of this species. It also suggests that TBM-based diets will be ecofriendly by reducing phosphorus load to the environment. These results would be supportive of the sustainable development of aquaculture for this commercially important species.

## 1. Introduction

Fish meal (FM) is considered a superior protein source in commercial aquafeeds, especially for carnivorous fish species. However, it has been a global issue in recent years to reduce dependency on FM owing to their unstable supply and high price. Although plant-based protein sources, such as soybean meal, rapeseed meal, and corn gluten meal, have been the focus of research because of their relatively low price and availability [1,2,3,4,5,6,7,8,9,10,11], several adverse effects due to the presence of anti-nutritional factors have also been reported in different species [8,10,11,12]. Moreover, the suitability of animal by-products and insect meals as an alternative to FM has been investigated in different species [13,14,15,16,17,18,19,20,21,22,23,24].

In addition, by-products from fish-processing industries, which comprise 30–80% of unprocessed fish body weight [25], have good nutritional value [26]. It is assumed that tuna by-products (heads, bones, skin, fins, and viscera) comprise about 52–54% of the total body weight of unprocessed tuna species [27]. Among fishery by-products, different types of tuna by-product meal (TBM) have been used to replace FM in several fish species. Examples are steam-dried TBM in spotted rose snapper, *Lutjanus guttatus* [28], and rockfish, *Sebastes schlegeli* [29]; fermented and non-fermented TBM in olive flounder, *Paralichthys olivaceus* [30,31,32]; tuna muscle by-product powder in olive flounder [33]; etc. The range of 12.5–75% replacement of FM with TBM from the above studies suggests that TBM is a promising protein source that needs to be determined for its application in other species.

The greater amberjack (*S. dumerili*), distributed across the tropical and subtropical regions of the Atlantic and Indo-Pacific Oceans, stands out for its rapid growth and high market value [34]. As a leading candidate species for aquaculture diversification, it has received increasing attention in recent years [35,36,37]. The greater amberjack shares morphological and ecological traits with the yellowtail (*Seriola quinqueradiata*), which ranks first in total annual production in Japan. Although the greater amberjack has significant potential as an important aquaculture species, reports on alternative protein sources in the diet of this species are limited [38], hindering the development of cost-effective feed and presenting a significant obstacle to maximizing its aquaculture potential. Although the suitability of TBM has been identified in various species, to the best of our knowledge, no report currently exists on this aspect in the greater amberjack. It has been assumed that the phosphorus (P) from an ingredient, which exists in the form of insoluble hydroxyapatite, is not well utilized by fish [39]. Since P is considered one of the reasons for eutrophication by contributing to the excessive growth of macrophytes and algae [40], an ingredient with a low content of P, such as TBM, in this study, would help reduce its discharge to the environment. Therefore, this study aimed to determine the optimal level of FM replacement based on growth performance, nutrient digestibility, body composition, and plasma parameters. Furthermore, it also investigated whether the discharge of P from TBM-based diets to the aquatic environment can be reduced.

## 2. Materials and Methods

### 2.1. Products, Dietary Formula, and Composition

The TBM used in this study was prepared by RegenWorks Co., Ltd. (Fukushima, Japan) from the by-products of yellowfin tuna (*Thunnus albacares*), bigeye tuna (*Thunnus obesus*), albacore (*Thunnus alalunga*), Pacific bluefin tuna (*Thunnus orientalis*), and southern bluefin tuna (*Thunnus maccoyii*). Raw materials (heads, bones, fins, blood, and skin) of the above-mentioned species were steam-cooked, treated with a mixture of peptidase and protease enzymes at a concentration of 0.1% (Proteax, Amano Enzyme Co., Ltd., Nagoya, Japan), dried at 60 °C for 20 h with a vacuum dryer (Vacuum separator FED-500, F·E·C Co., Ltd., Tokyo, Japan), and pulverized (Feather mill FM-1S, Hosokawa Micron Co., Ltd., Tokyo, Japan) after removing some of the bones to prepare the TBM powder. A comparison of the proximate composition, AA, free AA, and fatty acid (FA) content between the ingredients is provided in Table 1, Table 2, Table 3 and Table 4, respectively. The crude protein, crude fat, and mercury (Hg) contents were higher in TBM, but the ash, P, and calcium contents were higher in FM (Table 1). All indispensable (IAA) and dispensable (DAA) AA contents in TBM were higher than those in FM, with the exception of glycine (Table 2). When comparing free AAs, all free IAA and DAA contents, except histidine, were higher in TBM than in FM (Table 3). Palmitic acid (C16:0), palmitoleic acid (C16:1), stearidonic acid (C18:4n-3), and EPA (C20:5n-3) were remarkably higher in FM, but oleic acid (C18:1n-9), gadoleic acid (C20:1n-9), and linolenic acid (C18:3n-3) were higher in TBM (Table 4).

Table 5 shows the dietary formula and proximate composition of all experimental diets. The control diet (C) contained FM as the main protein source. The FM-derived protein contained in diet C was replaced at 25%, 50%, 75%, or 100% by TBM, and experimental diets were termed TBM25, TBM50, TBM75, and TBM100, respectively. To formulate the experimental diets, all ingredients were properly mixed, and fresh water was added to make dough. A laboratory pellet machine (12VR-750SDX, Alpha Royal Co., Ltd., Osaka, Japan) was used to pelletize the dough. The diets were then dried at 60 °C for 24 h to prepare the dry diet and stored at −20 °C until feeding. The crude protein and crude fat contents in the experimental diets ranged from 55.7 to 56.4% and 14.2 to 14.4%, respectively. However, although the P content tended to decrease with increasing levels of TBM, Hg contents rose with increasing levels of TBM in diets.

The AA and free AA compositions of the test diets are presented in Table 6 and Table 7, respectively. The content of all IAA, except histidine, increased with increasing amounts of TBM compared with the control diet. Regarding DAA, glutamic acid and tyrosine contents increased as the amount of TBM in the diets increased, whereas the glycine and serine contents decreased with increasing amounts of TBM (Table 6). The content of all free AAs, except histidine, tended to increase with increasing amounts of TBM in the diet (Table 7). Table 8 shows the FA compositions of the test diets. As the amount of TBM increased, the total monounsaturated FA content tended to increase, but the total saturated and polyunsaturated FA contents tended to decrease.

### 2.2. Fish, Husbandry, Samples, and Feces Collection

After purchasing 700 greater amberjack juveniles from A-marine Kindai (Shirahama, Japan), a venture company at Kindai University, Japan, the fish were transferred to an indoor research facility at the Aquaculture Research Institute at Uragami Station, Kindai University. Fish were stocked in a 3000 L circular indoor rearing tank, acclimatized for 1 month, and fed a commercial diet (approximately 52% crude protein; crude lipid: 12%, Marubeni Nisshin Feed Co., Ltd., Tokyo, Japan) twice daily (08:00 and 14:30) until apparent satiation.

The greater amberjack juveniles were fasted for 24 h and anesthetized using 250 ppm phenoxyethanol (Wako Pure Chemical Industries Ltd., Osaka, Japan) after the acclimation period. A group of 30 fish (mean weight: approximately 6.7 g) was randomly distributed in each of the fifteen 500 L circular black polyethylene tanks. The experiments were performed in triplicates for each treatment. Another 30 juveniles were randomly selected from the remaining fish and stored at −80 °C to determine the whole-body proximate composition of the initial fish. During the 42 d rearing period, the juveniles were fed twice daily until apparent satiation at 08:30 and 14:30, 6 d per week. The fish were cultured in a flow-through system, and the water flow rate was set at 7 L/min per tank. The photoperiod was 12 h of light (07:00–19.00) and 12 h of darkness, and water temperature and dissolved oxygen levels during the rearing period were 26.4 ± 1.1 °C and 5.7 ± 0.8 mg/L, respectively. The bottom was cleaned daily with a siphon at 11:00 a.m., and dead fish were counted and weighed if mortality occurred during the rearing period.

To observe progress, the fish in all tanks were weighed in pools at 2 and 4 weeks and at the end of the growth trial after being anesthetized with 300 ppm phenoxyethanol (Wako Pure Chemical Industries, Ltd., Osaka, Japan). At the end of the 42 d rearing period, five fish were sampled from each tank and frozen at −80 °C to determine the final whole-body proximate composition. Moreover, after collecting blood from another three fish from each tank, it was centrifuged at 3000× *g* for 15 min at 4 °C, and plasma was stored at −80 °C until the analysis. Blood collection was carried out from 9 am to 11 am. Another three fish from each tank were used to measure relative organ weights (viscera, liver, stomach, pyloric caeca, and intestine).

To determine the digestibility of the test diets, 10 fish from each of the three tanks for each treatment were pooled to stock 30 fish in a 350 L conical fecal collection tank, providing rearing conditions similar to those of the growth trial. The fish were fed the same experimental diets, after including 0.5% chromic oxide as an inert marker, and acclimatized to the fecal collection tanks for 1 week before fecal collection. During the fecal collection period, the fish were fed once daily at 15:00 until apparent satiation; the fecal collection column was thoroughly cleaned at approximately 1.5 h after feeding; the feces were collected at 08:30 the following day, and stored at −80 °C after removing excess water.

### 2.3. Biochemical Analyses and Calculation of Growth Parameters

To determine the proximate composition (moisture, protein, fat, and ash) of the diets, initial and final whole fish body, and fecal protein and fat, AOAC methods [41] were used. In brief, the moisture and ash content were determined at 100 °C and 550 °C, respectively, until a constant weight. Crude protein content was determined using an automatic Kjeldahl system (Kjeltec^TM^8400, Foss Analytical Co., Ltd., Suzhou, China), and crude fat content was determined by Soxhlet extraction using diethyl ether for 16 h. The chromic oxide content in the diet and feces was determined using the wet acid digestion method [42]. The P contents of the diets and whole bodies of the fish were determined according to Baginski et al. [43]. The method described by Folch et al. [44] was used to determine the fatty acid (FA) content of the experimental diets and fish whole bodies using a gas chromatograph (GC4000, GL Science, Tokyo, Japan) equipped with a capillary column (InterCap-Pure-WAX, GL Science). High-performance liquid chromatography (HPLC; GL7700, GL Science, Tokyo, Japan) was used to determine the amino acid (AA) content of the diets according to the method described by Teshima et al. [45]. Commercial kits (Fuji Dry-Chem, Fujifilm Company Ltd., Tokyo, Japan) were used to determine the plasma levels of total protein (TP), total cholesterol (TC), triglycerides (TGs), glutamic oxaloacetic transaminase (GOT), glutamic pyruvic transaminase (GPT), glucose (GLU), and albumin (ALB).

The weight gain (WG), specific growth rate (SGR), daily feed intake (DFI), feed efficiency (FE), condition factor (CF), survival rate, and somatic indices of the viscera (VSI), liver (HSI), stomach (SSI), pyloric caeca (PSI), and intestine (ISI); apparent digestibility coefficient (ADC) of protein and fat; productive values of protein (PVP), fat (PVF), and phosphorus (PVPs); and phosphorus intake, accumulation, and loading in the environment were calculated as follows.

WG (%) = 100 × (final mean weight − initial mean weight)/initial mean weight

SGR (%/day) = 100 × (ln final weight − ln initial weight)/rearing period (days)

DFI (g/100 g fish/day) = 100 × total feed intake/[(INF + FNF)/2 × (IW + FW)/2× rearing period],

where INF, FNF, IW, and FW indicate the initial number of fish, final number of fish, initial weight, and final weight, respectively.

FE (%) = 100 × [total wet weight gain (g)/total dry feed intake (g)]

CF = 1000 × (W/L^3^),

where W is the wet body weight (g) and L is the fork length (cm).

Survival rate (%) = 100 × final number of fish/initial number of fish in a tank

VSI, HSI, SSI, PSI and ISI (%) = 100 × [wet weight of viscera, liver, stomach, pyloric caeca and intestine (g)/wet body weight (g)]

ADC of protein or fat (%) = 100 × [1 − {(dietary Cr_2_O_3_/fecal Cr_2_O_3_) × (fecal protein or fat/dietary protein or fat)}]

PVP, PVF, and PVPs (%) = 100 × [(final fish body protein, fat and phosphorus − initial fish body protein, fat and phosphorus)/total protein, fat and phosphorus intake]

Phosphorus intake (g/kg body weight gain) = total phosphorus in consumed diet (g)/body weight gain in kg

Phosphorus accumulation (g/kg body weight gain) = {total final fish body phosphorus (g) − total initial fish body phosphorus (g)}/body weight gain in kg

Phosphorus loading (g/kg body weight gain) = {total phosphorus intake through diet (g) − total phosphorus deposited into body (g)}/body weight gain in kg

### 2.4. Statistical Analysis

Statistical analyses were conducted using the SPSS program for Windows (v. 10.0). Data are expressed as the mean ± standard deviation of triplicate samples, except for relative organ weights (n = 9). The Kolmogorov–Smirnov test was used to check the normality of the data, and the homogeneity of variance was also checked using the Levene statistic in the SPSS program. Mean comparisons between treatments were performed using a one-way analysis of variance (ANOVA). In instances where differences were detected by ANOVA, Tukey’s test was used to rank the treatments at *p* < 0.05. The optimal level of TBM in the diet was determined using a quadratic polynomial regression analysis.

## 3. Results

### 3.1. Growth Performance and Relative Organ Weight

**Figure 1:**
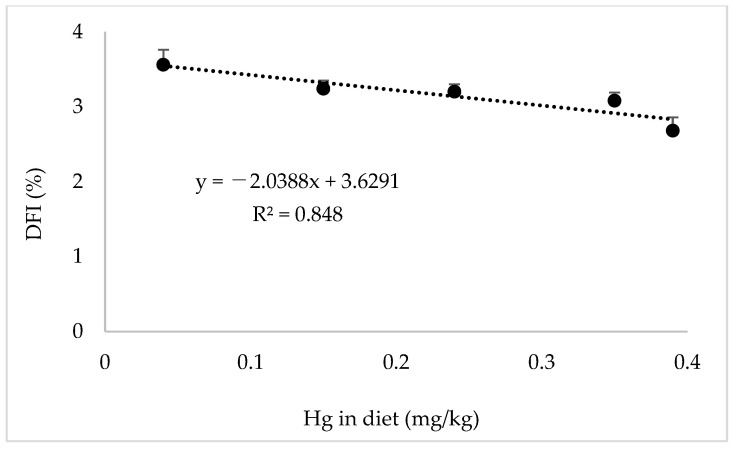
Linear correlation between Hg content in the diet and daily feeding rate.

**Figure 2:**
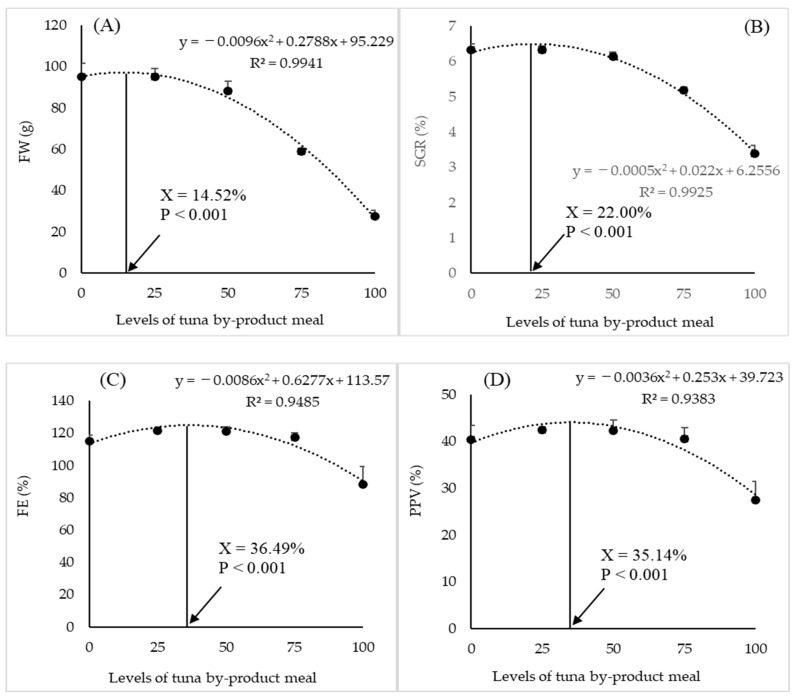
The quadratic polynomial regression analysis of the final weight (**A**), specific growth rate (**B**), feed efficiency (**C**), and productive value of protein (**D**) against the dietary levels of the TBM. The arrow indicates the maximum level of FM substitution for TBM in the diet of juvenile greater amberjack.

Table 9 shows growth performance and biometric indices at the end of the rearing period. Although there were no significant differences in FMW, WG, and SGR between fish fed with diets C and TM25, the FMW, WG, and SGR of fish fed diets TM50, TM75, and TM100 were significantly lower than those of fish fed the C diet (*p* < 0.05). The DFI ranged from 3.56 to 2.68 g/100 g fish/day and showed a decreasing trend with increasing TBM inclusion levels in the diets, and that of fish fed the TM50, TSM75, and TM100 diets was significantly lower than that of fish fed diet C (*p* < 0.05). However, the FE was significantly lower in the TM100 diet group than in the control group (*p* < 0.05). There were no significant differences in the survival rate, CF, or relative organ weights (VSI, HSI, PSI, SSI, and ISI, *p* > 0.05). A significant negative linear correlation was found between dietary Hg content and DFI (Figure 1, R^2^ = 0.848). From the quadratic polynomial regression analysis, the optimal dietary levels of TBM for final weight, SGR, FE, and PPV were 14.5, 22.0, 36.5%, and 35.1%, respectively (Figure 2).

### 3.2. Whole-Body Proximate Composition and Fatty Acid Composition

**Figure 3:**
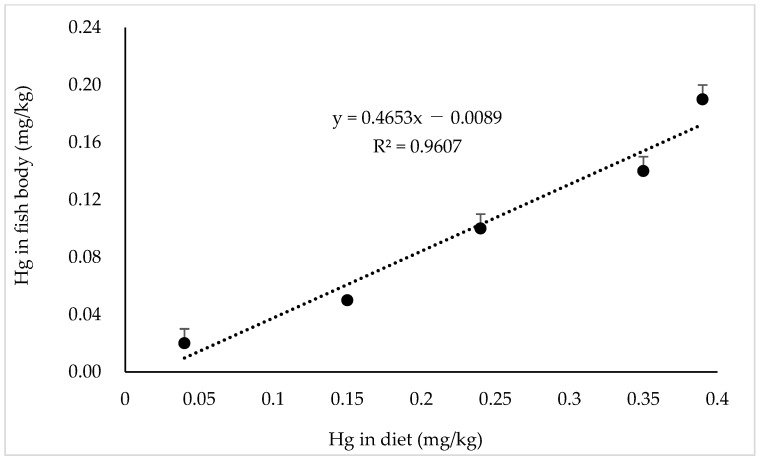
Linear correlation between Hg content in diet and fish whole body.

The whole-body proximate compositions and Hg contents in the fish at the experiment’s initial and final stages are listed in Table 10. For proximate composition, moisture, crude protein, and crude fat contents in fish fed the diet TM100 showed significant differences compared to the other diets (*p* < 0.05). However, there was no significant difference in the ash content between the treatments (*p* > 0.05). The final whole-body Hg content tended to increase significantly with increasing levels of TBM in the diets (*p* < 0.05), resulting in a significant linear correlation between Hg in diets and whole-body Hg in the fish (Figure 3, R^2^ = 0.9607).

The final whole-body FA composition is shown in Table 11. A decreasing trend was observed in the final whole body of C14:0 (myristic acid), C16:1 (palmitoleic acid), C20:1n-9 (gadoleic acid), C18:4n-3 (stearidonic acid), and C20:5n-3 (eicosapentaenoic acid) with increasing levels of TBM in the diets, and those in diets TM50, TM75, and TM100 were significantly lower than those in the control group (*p* < 0.05). In contrast, the final whole-body C18:0 (stearic acid), C18:1n-9 (oleic acid), C18:2n-6 (linoleic acid), C20:4n-6 (arachidonic acid), and C22:5n-6 (docosapentaenoic acid) levels showed an increasing trend with increasing TBM levels in the diets, being those in the TM50, TM75, and TM100 groups that were significantly higher than those in the control group (*p* < 0.05). However, the other FAs did not differ significantly among the treatments (*p* > 0.05).

### 3.3. Nutrient Digestibility and Productive Value

Table 12 shows nutrient digestibility and productivity values. There were no significant differences in the digestibility of protein and fat between the treatments (*p* > 0.05). Neither were there significant differences in protein and fat productive values among the fish fed diets C, TM25, TM50, and TM75, but those of fish fed diet TM100 were significantly lower compared to other diets (*p* < 0.05). The P productive value showed a rising trend with increasing inclusion levels of TBM in diets, and fish fed diets TM75 and TM100 showed significantly higher values compared to the control diet (*p* < 0.05).

### 3.4. Phosphorus Budget

The intake, accumulation, and loading rates of P per kilogram of weight gain are listed in Table 13. P intake and loading rates per kilogram weight gain showed a decreasing trend with increasing levels of TBM in the diets, where all TBM-based diets showed significantly lower values than those of fish fed the control diet (*p* < 0.05). However, P accumulation did not vary significantly among treatments (*p* > 0.05).

### 3.5. Plasma Analysis

The plasma parameters of greater amberjack are listed in Table 14. TP, ALB, and TC showed similar patterns among the treatments, and the parameters in the TM75 and TM100 diets were significantly lower than those in the C, TM25, and TM50 diets (*p* < 0.05). Although there was no specific trend in TG content among the treatments, diet TM75 showed a significantly higher value than the control diet (*p* < 0.05). However, there were no significant differences in GOT, GPT, or GLU contents among the treatments (*p* > 0.05).

## 4. Discussion

In this study, the quadratic polynomial regression analysis revealed that the optimal dietary levels of TBM for final weight, SGR, FE, and PRE were 14.5, 22.0, 36.5%, and 35.1%, respectively, suggesting that FM protein can be replaced at least at 14.5% with TBM without compromising growth performance in juvenile greater amberjacks. Early studies on the replacement of FM with TBM with or without fermentation have shown a wide range of FM replacements in different species. When TBM without fermentation was used, it was demonstrated that FM replacement levels were 25–30% in the juvenile spotted rose snapper [28]; 30–75% in the Korean rockfish, *Sebastes schlegeli* [29]; and 50% in the olive flounder [32]. In olive flounder, the tuna muscle by-product powder can replace 50% of FM protein without affecting growth [33]. However, when fermented TBM was used, only 10.6–20.6% of the FM protein could be replaced in olive flounder [30]. In the present study, TBM, which was treated with protease enzymes during processing as mentioned earlier, could replace at least 14.5% of the FM protein in the diet of juvenile greater amberjack. It is difficult to compare the levels of FM replacement between studies since the proportion of FM in the control diet is not the same in different studies. However, this difference in the substitute level of dietary FM by TBM may be due to product types, nutritional composition (protein, amino acids, etc.), digestibility of products, rearing condition, and fish species and sizes.

The plausible causes of low DFI, as well as poor growth in different species, and the relevance of these issues in this study are discussed onward. First, one of the most important reasons for poor growth in fish from alternative protein sources is assumed to be the low DFI that could be caused by reduced palatability. Although the palatability was not directly measured, a significant reduction in DFI from 3.56 g/100 g fish/day in diet C to 2.68 g/100 g fish/day in diet TM100 suggests that the acceptability of the TBM-based diet was somehow affected. It is assumed that the reduced DFI may be mediated through the presence of a component with an aversive taste or an imbalance in the composition of AA or free AAs. For example, a reduction in growth performance was reported in juvenile Nile tilapia, *Oreochromis niloticus*, due to an imbalance of IAA in the waste from the tuna industry [46]. In olive flounder, growth performance differed due to differences in DFI associated with low free AA content in the tuna muscle by-product meal [33]. However, all IAAs and free IAAs, except histidine, in the TBM or TBM-based diets were higher than those in the control diet. Although information on IAA requirements in greater amberjack is scarce, it has been shown that the dietary lysine requirements of greater amberjack juveniles range from 2.03 to 2.11% [47], and lysine levels in the TBM-based diets range from 3.98 to 4.69%. Histidine has been reported to have highly aversive taste stimuli in fish [48], and increasing dietary levels affect DFI and growth in olive flounder [33]. However, histidine or free histidine levels in TBM or TBM-based diets were similar or lower than those in the control diet, suggesting that IAA is unlikely to be the reason for poor growth in TBM-based diets, in which more than 50% of FM protein was replaced by TBM. It has been demonstrated that undesirable taste is one of the shortcomings of the successful utilization of certain by-products [49]. It has also been suggested that some by-product production procedures may lead to a negative change in the peptide structure and reduce the availability of protein sources in fish diets [50]. Therefore, more studies are necessary to clarify whether these issues are involved in the low availability of TBM.

Second, for fast-growing marine carnivorous species, long-chain polyunsaturated FAs (LC-PUFAs), such as arachidonic acid (C20:4n-6), eicosapentaenoic acid (C20:5n-3, EPA), and docosahexaenoic acid (C226n-3, DHA), are considered important for maintaining fast growth, FE, and survival, as well as for maintaining health status [51]. The information on the nutritional requirements of greater amberjacks remains scarce. However, when fish oil in the diet of juvenile greater amberjack was replaced with vegetable oil blends, it was suggested that levels of n-3 LC-PUFA up to 12 g/kg diet could meet the essential fatty acid (EFA) requirements of this species [52]. Consequently, it was also suggested that the recommended levels of n-3 PUFA in the greater amberjack larvae at the feeding stage of *Artemia* and in the broodstock diet were 12–17% of total fatty acid [53] and 1.0–1.7 g/100 g diet [37], respectively. In the present study, the lowest level of n-3 PUFA was 24.1% of the total fatty acid diet TM100. When this level is converted to total dietary fatty acid, according to Yoshimatsu et al. [54], it is equivalent to 30.5 g/kg diet or 3.05 g/100 g diet (fatty acid, % in diet = total dietary lipid × 0.892). Therefore, dietary n-3 LC-PUFA may not be directly related to the lower growth performance of TBM-based diets. However, an overall balance between energy and essential FA requirements (saturated, monounsaturated, and polyunsaturated fatty acids) is required, particularly in rapidly growing fish [55]. In this study, the balance between energy and essentiality may not have been maintained, as the dietary oleic acid (C18:1n-9) was higher and the EPA content was lower in TBM-based diets. Since EPA is considered an important essential FA as mentioned above, it is necessary to investigate whether a decreasing trend in EPA content in TBM-based diets affected fish growth. It has also been suggested that the requirement for any given FA is determined not only by its absolute amount but also by its ratio with other FAs [55]. For example, when fish oil was replaced with vegetable oil in the diet of juvenile greater amberjacks, there was no significant difference in growth performance when the DHA/EPA and EPA/ARA ratios were similar between the diets [52]. Although the ratio of DHA/EPA gradually increased with increasing amounts of TBM in this study, the ratio of EPA/ARA decreased and remained less than half in diets in which more than 50% of the FM protein was replaced. Therefore, further investigations are necessary to determine whether the balance between FAs is responsible for the poor growth observed in the present study. In general, the FA composition of the diet was reflected in the final whole-body weight of greater amberjack juveniles.

Third, when TBM is used in fish diets, the presence of heavy metals is a concern regarding its effect on growth as well as its impact on consumer health [56]. It was found that the exposure of Atlantic salmon, *Salmo salar*, to waterborne inorganic Hg and organic methylmercury (MeHg) affected the olfactory system [57]. However, when Atlantic salmon parr was orally exposed to both Hg and MeHg, sensory behavior was not affected during 4 months of exposure [58]. Similar results were also found in the yellowfin bream, *Acanthopagrus australis* [59]; however, a decrease in appetite was observed in zebrafish, *Danio rerio*, after 47 d of oral exposure [60]. In this study, there was a significant negative linear correlation between dietary Hg levels and DFI. The negative correlation may suggest that the dietary Hg content may affect the appetite in fish fed TBM-based diets, as was observed in zebrafish. However, the experimental design does not confirm that the reduction in DFI is solely due to the Hg content in the diet. Therefore, further research is necessary to elucidate whether the Hg level in the diet has a negative effect on feed acceptance in greater amberjack.

Furthermore, there was a significant linear correlation between the dietary Hg content and whole-body Hg levels in fish. In Japan, hundreds of people surrounding Minamata Bay and Niigata were affected by Minamata Disease (a nervous system disorder) in the 1950s and the 1960s, which resulted from the consumption of highly MeHg-contaminated fish from industrial sources [61]. Fish are the main source of Hg, and approximately 80–90% of that consumed is derived from fish and other marine products [62]. The recommendations for the Provisional Tolerable Weekly Intake (PTWI) proposed by the Food and Agriculture Organization (FAO) and World Health Organization (WHO) expert committee are 3.3 μg MeHg/kg body weight for the general population and 1.6 μg MeHg/kg body weight to protect a developing fetus [63]. In the present study, the total whole-body Hg content was determined, and the highest value was 0.19 ± 0.01 mg/kg (0.19 ± 0.01 μg/g) body weight in greater amberjack. Although MeHg was not determined in the muscle of greater amberjack as the fish was notably smaller compared to the commercial size, it was found that only 0.67–1.60% of ingested Hg is converted to MeHg in the fish intestine by bacterial activity [59], suggesting that the level of MeHg in greater amberjack would be lower. Moreover, the total Hg level in this study is considered to be far lower than the reported levels in the muscle of some popular tuna species, such as 0.42 ± 0.06 μg/g in Atlantic bluefin tuna, *Thunnus thynnus*; 0.59 ± 0.34 μg/g in Pacific bluefin tuna, *T. orientalis*; and 0.98 ± 0.34 μg/g in bigeye tuna, *T. obesus* [63]. However, it is necessary to determine the Hg levels when fish are fed a TBM-based diet until they reach a marketable size.

Except for the ash content, the final whole-body proximate composition of the juvenile greater amberjack was affected by the experimental diets. As discussed by Jobling [64], a strong negative correlation was found between whole-body moisture and fat content in this study, where the former increased with increasing levels of TBM in the diet, and the latter showed the opposite trend. Both crude protein and crude fat contents showed the same trend among the treatments. Although the DFI was decreased in fish fed the TM25, TM50, and TM75 diets compared to the control diet, the lack of significant differences in protein and fat digestibility and productive values between those diets may have helped to maintain a similar whole-body composition of these components. There were no significant differences in the digestibility of protein and fat in fish fed the diet TM100 compared to the other diets; however, the productive value of both parameters was significantly lower in fish fed the diet TM100. The lower amount of feed due to the lower DFI in diet TM100 may allow the fish to digest properly, resulting in digestibility similar to that found in other diets. However, a significantly lower final whole-body crude protein and crude fat content and productive value of both protein and fat in diet TM100 may be because of the insufficient assimilation of those nutrients from a lower DFI, resulting in poor deposition in fish. When TBM is used to replace FM, its effect on the whole-body proximate composition of fish has been reported in some studies [33], but not in others [29,30,32]. The discrepancy between studies may be attributed to the dietary formula, fish species and size, rearing temperature, and feeding behavior.

As whole-body P accumulation in fish was similar among the treatments, a gradual decrease in P intake from TBM-based diets resulted in a significantly lower P load in the environment. It has been found that the existence of P in the form of insoluble hydroxyapatite in FM, which comes from hard tissues (bones and scales), makes it less utilizable [65]. In the present study, no statistically significant differences in growth performance were observed between fish fed diets C and TM25; however, the P load from fish fed diet TM25 was approximately 17% lower than that of the FM-based control diet, suggesting that TBM-based diets could be made more environmentally friendly by reducing P discharge into the environment. Similarly, a 50% reduction in P load was reported in olive flounder when 50% of FM protein was replaced by a by-product of tuna muscle [33]. Moreover, a reduction in P load was also reported in European sea bass, *Dicentrarchus labras* [66], where plant proteins or different combinations of protein ingredients were used.

Blood parameters are considered important indicators of fish health when FM is replaced by other protein sources. Although there were no significant differences in the plasma levels of GOT and GPT, which are used to evaluate liver function and are associated with liver necrosis [67], the TP content was significantly reduced when >75% of the FM protein was replaced by TBM. Although there was no significant difference in TP content when up to 50% of FM protein was replaced in the diet of olive flounder by either fermented TBM [30] or unfermented TBM [32], a higher replacement level affected the plasma level of TP in the same species [68], which is consistent with this study. ALB, a family of globular proteins that constitutes approximately half of the serum protein but differs from other blood proteins in that they are not glycosylated, also showed a similar pattern to that of TP. Because ALB is known to be involved in transporting hormones, FAs, and other compounds [69], a significant reduction in ALB at higher replacement levels in this study may have affected fish growth. Plasma levels of TC, which are important for maintaining the levels of bile acids involved in the digestion and absorption of lipids, were also significantly affected at higher replacement levels in this study. A similar result was observed in olive flounder when FM was replaced with fermented TBM [30] but differed from other studies using unfermented TBM [32]. Although the plasma levels of TG showed significant differences between treatments, there was no specific trend in the levels of TBM in the diets.

## 5. Conclusions

Although growth performance and plasma parameters did not differ significantly until 25% of the FM protein was replaced by TBM, the polynomial regression analysis suggested that at least 14.5% of the FM protein could be replaced by the TBM protein in the diet of juvenile greater amberjacks, at least in the size and rearing conditions used here. Furthermore, the discharge of P to the environment can be reduced by 17% when 25% of the FM protein is replaced with TBM. The increasing level of the total body Hg content suggests that it is necessary to assess the Hg/MeHg levels when fish are fed a TBM-based diet until they reach a marketable size to determine the health risk to people. Further studies are also necessary to determine the reasons for the lower growth performance with increasing TBM levels in the diet.

## Figures and Tables

**Table 1:** Proximate composition of fish meal and tuna by-product meal.

Parameters	Fish Meal	Tuna By-Product Meal
Crude protein (%)	70.4	75.2
Crude fat (%)	10.2	21.1
Ash (%)	16.7	1.6
Phosphorus (%)	2.42	0.32
Calcium (%)	3.23	0.21
Mercury (mg/kg)	0.03	0.59

**Table 2:** Amino acid composition (g/100g diet, dry basis) of fish meal and tuna by-product meal.

Ingredients	Fish Meal	Tuna By-Product Meal
Indispensable amino acids (IAAs)		
Arginine	3.66	4.59
Histidine	2.09	2.10
Isoleucine	2.64	3.88
Leucine	4.63	6.29
Lysine	5.06	7.11
Methionine	1.78	2.37
Phenylalanine	2.61	2.89
Threonine	2.70	3.37
Tryptophan	0.80	0.85
Valine	3.14	4.17
ΣIAA	29.1	37.6
Dispensable amino acids (DAAs)		
Alanine	3.66	4.68
Aspartic acid	5.79	7.38
Cystine	0.63	0.68
Glutamic acid	8.10	11.50
Glycine	3.93	3.45
Proline	2.50	2.59
Serine	2.45	2.66
Tyrosine	2.03	2.76
ΣDAA	29.1	35.7

**Table 3:** Free amino acid composition (mg/100g diet, dry basis) of fish meal and tuna by-product meal.

Ingredients	Fish Meal	Tuna By-Product Meal
Indispensable amino acids (IAAs)		
Arginine	101	1320
Histidine	663	600
Isoleucine	62	1350
Leucine	124	3010
Lysine	117	1780
Methionine	3	950
Phenylalanine	59	1180
Threonine	65	610
Tryptophan	13	190
Valine	86	1380
ΣIAA	1293	12,370
Dispensable amino acids (DAAs)		
Alanine	211	1210
Aspartic acid	32	420
Cystine	0	0
Glutamic acid	113	1020
Glycine	59	220
Proline	55	140
Serine	42	170
Tyrosine	56	720
ΣDAA	568	3900

**Table 4:** Fatty acid composition (% of total fatty acids) of fish meal and tuna by-product meal.

Fatty Acids	Fish Meal	Tuna By-Product Meal
C14:0	6.8	2.2
C15:0	0.5	0.4
C16:0	20.1	14.9
C18:0	4.4	5.1
ΣSFA	31.8	22.6
C16:1	7.1	3.1
C18:1n-9	10.1	27.7
C20:1n-9	0.4	2.6
ΣMUFA	17.6	33.4
C18:2n-6	0.1	0.7
C20:3n-6	0.1	0.3
C20:4n-6	1.1	1.4
C22:5n-6	0.4	1.0
Σn-6	1.7	3.4
C18:3n-3	0.7	2.4
C18:4n-3	2.1	0.7
C20:4n-3	0.5	0.5
C20:5n-3 (EPA)	13.9	4.4
C22:5n-3	1.6	1.8
C22:6n-3 (DHA)	19.3	17.9
Σn-3	38.1	27.7
ΣPUFA	39.8	31.1

**Table 5:** Formula and proximate composition of diets used to feed greater amberjack.

Ingredients (%)	C	TM25	TM50	TM75	TM100
Fish meal ^1^	70.0	52.5	35.0	17.5	0.0
Tuna meal	0.0	16.3	32.7	49.0	65.3
Fish oil ^2^	7.0	5.3	3.6	2.0	0.3
Wheat flour ^3^	10.0	10.0	10.0	10.0	10.0
α-Starch	8.0	8.0	8.0	8.0	8.0
Vitamin mix ^4^	2.0	2.0	2.0	2.0	2.0
Mineral mix ^4^	2.0	2.0	2.0	2.0	2.0
Stay-C 35	0.2	0.2	0.2	0.2	0.2
Calcium phosphate	0.4	1.0	1.2	1.5	1.7
Cellulose	0.4	2.7	5.3	7.8	10.5
Proximate composition (%, dry basis)					
Crude protein	55.9	56.4	56.1	55.8	55.7
Crude fat	14.4	14.4	14.3	14.3	14.2
Ash	14.3	11.9	9.3	6.7	4.6
Phosphorus (g/kg)	19.4	18.8	15.9	12.5	9.7
Mercury (mg/kg)	0.04	0.15	0.24	0.35	0.39

^1^ TASA, Lima, Peru (crude protein, ca. 70%; crude lipid, ca. 10%). ^2^ Tsuji Oil Co., Ltd. (Tokyo, Japan). ^3^ Nisshin Flour Milling Inc., Tokyo, Japan. ^4^ Marubeni Nisshin Feed Co., Ltd. (Tokyo, Japan) formula.

**Table 6:** Amino acid composition (g/100g diet, dry basis) of experimental diets.

Diets	C	TM25	TM50	TM75	TM100
Indispensable amino acids (IAAs)					
Arginine	2.81	3.01	3.09	3.14	3.06
Histidine	1.60	1.62	1.59	1.48	1.45
Isoleucine	2.02	2.27	2.49	2.59	2.63
Leucine	3.56	3.91	4.20	4.30	4.32
Lysine	3.59	3.98	4.36	4.53	4.69
Methionine	1.24	1.25	1.37	1.39	1.49
Phenylalanine	2.00	2.07	2.06	2.05	2.05
Threonine	2.08	2.08	2.18	2.09	2.21
Tryptophan	0.61	0.63	0.62	0.62	0.62
Valine	1.24	2.61	2.79	2.83	2.85
ΣIAA	20.8	23.4	24.8	25.0	25.4
Dispensable amino acids (DAAs)					
Alanine	3.02	3.20	3.27	3.23	3.13
Aspartic acid	4.43	4.63	4.90	4.80	4.96
Cystine	0.50	0.48	0.48	0.44	0.45
Glutamic acid	6.48	7.14	7.69	7.93	8.06
Glycine	2.93	2.92	2.8	2.58	2.38
Proline	1.99	2.05	2.03	1.89	1.88
Serine	1.96	1.95	1.95	1.82	1.83
Tyrosine	1.50	1.63	1.73	1.75	1.76
ΣDAA	22.8	24.0	24.9	24.4	24.5

**Table 7:** Free amino acid composition (g/100g diet, dry basis) of experimental diets.

Diets	C	TM25	TM50	TM75	TM100
Indispensable amino acids (IAAs)					
Arginine	0.07	0.27	0.47	0.66	0.86
Histidine	0.46	0.45	0.43	0.41	0.39
Isoleucine	0.04	0.25	0.46	0.67	0.88
Leucine	0.09	0.56	1.03	1.50	1.97
Lysine	0.08	0.35	0.62	0.89	1.16
Methionine	0.02	0.16	0.31	0.47	0.62
Phenylalanine	0.04	0.22	0.41	0.59	0.77
Threonine	0.05	0.13	0.22	0.31	0.40
Tryptophan	0.01	0.04	0.07	0.10	0.12
Valine	0.06	0.27	0.48	0.69	0.90
ΣIAA	0.92	2.70	4.50	6.29	8.08
Dispensable amino acids (DAAs)					
Alanine	0.15	0.31	0.47	0.63	0.79
Aspartic acid	0.02	0.09	0.15	0.21	0.27
Cystine	0.00	0.00	0.00	0.00	0.00
Glutamic acid	0.08	0.23	0.37	0.52	0.67
Glycine	0.04	0.07	0.09	0.12	0.14
Proline	0.04	0.05	0.07	0.08	0.09
Serine	0.03	0.05	0.07	0.09	0.11
Tyrosine	0.04	0.15	0.26	0.36	0.47
ΣDAA	0.40	0.93	1.47	2.01	2.55

**Table 8:** Fatty acid composition (% of total fatty acids) of experimental diets.

Fatty Acids	C	TM25	TM50	TM75	TM100
C14:0	4.7	4.1	3.5	3.0	2.2
C15:0	0.4	0.4	0.4	0.4	0.4
C16:0	15.7	15.6	15.6	15.3	15.2
C18:0	3.4	3.8	4.3	4.6	5.0
ΣSFA	24.2	23.9	23.8	23.3	22.8
C16:1	5.5	5.0	4.4	3.9	3.2
C18:1n-9 (OA)	13.8	16.0	19.1	22.0	25.5
C20:1n-9	6.1	5.4	4.7	3.9	2.8
ΣMUFA	25.4	26.4	28.2	29.8	31.5
C18:2n-6	3.4	4.3	5.9	7.6	9.9
C20:3n-6	0.1	0.2	0.2	0.1	0.2
C20:4n-6 (ARA)	0.8	1.0	1.2	1.3	1.5
C22:5n-6	0.4	0.6	0.8	0.8	1.0
Σn-6	4.7	6.1	8.1	9.8	12.6
C18:3n-3	0.9	1.1	1.4	1.8	2.2
C18:4n-3	2.2	1.8	1.4	1.1	0.8
C20:4n-3	0.6	0.6	0.6	0.6	0.5
C20:5n-3 (EPA)	11.5	10.0	8.2	6.8	4.8
C22:5n-3	1.5	1.6	1.7	1.7	1.8
C22:6n-3 (DHA)	14.4	14.9	15.0	14.2	14.0
Σn-3	31.1	30.0	28.3	26.2	24.1
ΣPUFA	35.8	36.1	36.4	36.0	36.7
DHA/EPA	1.3	1.5	1.8	2.1	2.9
OA/DHA	1.0	1.1	1.3	1.5	1.8
EPA/ARA	14.4	10.0	6.8	5.2	3.2

**Table 9:** Growth performance and biometric indices in greater amberjack fed with different diets for 6 weeks.

Parameters	C	TM25	TM50	TM75	TM100
Growth performance					
IMW (g)	6.66 ± 0.02	6.66 ± 0.00	6.66 ± 0.01	6.67 ± 0.01	6.65 ± 0.01
FMW (g)	95.2 ± 6.5 ^a^	95.1 ± 3.9 ^a^	78.1 ± 4.8 ^b^	58.9 ± 1.4 ^c^	27.6 ± 2.9 ^d^
Survival rate (%)	93.3 ± 11.5	90.0 ± 8.8	91.1 ± 8.4	92.2 ± 13.5	63.3 ± 29.1
WG (%)	1329.3 ± 95.2 ^a^	1328.1 ± 58.4 ^a^	1073.4 ± 70.0 ^b^	782.9 ± 21.7 ^c^	315.5 ± 43.2 ^d^
SGR (%/day)	6.33 ± 0.16 ^a^	6.33 ± 0.17 ^a^	5.86 ± 0.14 ^b^	5.19 ± 0.06 ^c^	3.38 ± 0.25 ^d^
DFI (g/100 g fish/day)	3.56 ± 0.20 ^a^	3.24 ± 0.11 ^ab^	3.20 ± 0.10 ^bc^	3.08 ± 0.11 ^cd^	2.68 ± 0.18 ^d^
FE (%)	115.0 ± 4.1 ^a^	121.7 ± 1.1 ^a^	121.3 ± 2.4 ^a^	117.3 ± 3.2 ^a^	88.3 ± 11.0 ^b^
Biometric indices					
CF	16.0 ± 0.5	16.4 ± 0.9	16.1 ± 0.6	16.3 ± 0.9	16.2 ± 1.0
VSI	5.87 ± 0.70	6.14 ± 0.50	6.77 ± 0.39	6.69 ± 0.37	6.21 ± 0.34
HSI	0.72 ± 0.25	0.90 ± 0.11	0.91 ± 0.16	1.10 ± 0.11	1.05 ± 0.20
PSI	1.40 ± 0.23	1.48 ± 0.19	1.55 ± 0.21	1.53 ± 0.15	1.55 ± 0.29
SSI	1.10 ± 0.24	1.18 ± 0.14	1.33 ± 0.22	1.14 ± 0.24	1.17 ± 0.24
ISI	0.71 ± 0.17	0.79 ± 0.12	0.86 ± 0.18	0.73 ± 0.14	0.77 ± 0.19

Abbreviations: control, C; TM25, TM50, TM75, and TM100 are diets replacing 25, 50, 75, and 100% of fish meal protein by tuna by-product meal; IMW, initial mean weight; FMW, final mean weight; WG, weight gain; SGR, specific growth rate; DFI, daily feed intake; FE, feed efficiency; CF, condition factor; VSI, viscerosomatic index; HSI, hepatosomatic index; PSI, pyloric caeca somatic index; SSI, stomatosomatic index; ISI, intestinosomatic index. Values are mean ± SD of three replicate samples. Means in row with different superscripts are significantly different (*p* < 0.05, Tukey’s test).

**Table 10:** Whole-body proximate composition and mercury content of greater amberjack fed the experimental diets for 6 weeks.

Parameters	Initial	Final				
		C	TM25	TM50	TM75	TM100
Moisture (%)	81.2	73.0 ± 1.9 ^a^	73.4 ± 1.1 ^a^	72.3 ± 1.4 ^a^	71.9 ± 2.6 ^a^	78.3 ± 0.6 ^b^
Crude protein (%)	14.6	18.3 ± 0.7 ^a^	18.3 ± 0.2 ^a^	18.0 ± 0.5 ^a^	17.9 ± 0.6 ^a^	15.8 ± 0.4 ^b^
Crude fat (%)	2.3	6.1 ± 1.0 ^a^	5.7 ± 0.6 ^a^	6.3 ± 0.2 ^a^	6.3 ± 0.4 ^a^	3.1 ± 0.4 ^b^
Ash (%)	3.1	2.8 ± 0.7	2.9 ± 0.4	3.2 ± 0.3	3.5 ± 0.4	3.2 ± 0.3
Hg (mg/kg)	0.02	0.02 ± 0.01 ^a^	0.05 ± 0.00 ^b^	0.10 ± 0.01 ^c^	0.14 ± 0.01 ^d^	0.19 ± 0.01 ^e^

Values are mean ± SD of three replicate samples. Means in row with different superscripts are significantly different (*p* < 0.05, Tukey’s test).

**Table 11:** Fatty acid composition (% of total fatty acids) of initial and final whole body.

Fatty Acids	Initial	Final				
		C	TM25	TM50	TM75	TM100
C14:0	5.1	4.2 ± 0.1 ^a^	3.8 ± 0.0 ^a^	3.0 ± 0.1 ^b^	2.5 ± 0.0 ^bc^	1.9 ± 0.0 ^c^
C15:0	0.3	0.4 ± 0.0	0.4 ± 0.0	0.4 ± 0.0	0.4 ± 0.0	0.4 ± 0.0
C16:0	19.8	16.5 ± 0.2	16.4 ± 0.1	16.0 ± 0.1	15.7 ± 0.2	15.8 ± 0.2
C18:0	5.3	4.4 ± 0.0 ^a^	4.7 ± 0.1 ^ab^	5.2 ± 0.2 ^b^	5.5 ± 0.1 ^bc^	6.2 ± 0.1 ^c^
ΣSFA	30.5	25.5 ± 0.2	25.4 ± 0.1	24.7 ± 0.1	24.2 ± 0.2	24.3 ± 0.3
C16:1	4.6	5.7 ± 0.1 ^a^	5.3 ± 0.1 ^ab^	4.5 ± 0.0 ^bc^	4.0 ± 0.0 ^cd^	3.1 ± 0.1 ^d^
C18:1n-9 (OA)	19.0	16.9 ± 0.1 ^a^	18.4 ± 0.1 ^b^	21.5 ± 0.1 ^c^	24.8 ± 0.3 ^d^	25.7 ± 0.1 ^e^
C20:1n-9	2.4	6.1 ± 0.1 ^a^	5.6 ± 0.0 ^ab^	4.3 ± 0.1 ^b^	3.4 ± 0.1 ^c^	2.4 ± 0.1 ^d^
ΣMUFA	26.0	28.7 ± 0.1	29.3 ± 0.1	30.4 ± 0.2	32.2 ± 0.2	31.2 ± 0.1
C18:2n-6	3.0	4.0 ± 0.1 ^a^	4.7 ± 0.1 ^ab^	6.5 ± 0.0 ^b^	8.3 ± 0.2 ^c^	9.7 ± 0.3 ^d^
C20:3n-6	0.2	0.1 ± 0.0	0.1 ± 0.0	0.2 ± 0.1	0.3 ± 0.0	0.3 ± 0.0
C20:4n-6 (ARA)	0.9	0.8 ± 0.0 ^a^	0.9 ± 0.0 ^a^	1.2 ± 0.1 ^b^	1.3 ± 0.1 ^bc^	1.8 ± 0.1 ^c^
C22:5n-6	0.3	0.4 ± 0.0 ^a^	0.5 ± 0.0 ^a^	0.8 ± 0.0 ^b^	0.8 ± 0.1 ^b^	1.1 ± 0.0 ^c^
Σn-6	4.4	5.3 ± 0.1	6.3 ± 0.1	8.6 ± 0.1	10.7 ± 0.1	12.9 ± 0.2
C18:3n-3	1.1	1.0 ± 0.0	1.1 ± 0.1	1.4 ± 0.0	1.6 ± 0.1	1.7 ± 0.1
C18:4n-3	2.2	1.8 ± 0.0 ^a^	1.5 ± 0.1 ^ab^	1.2 ± 0.0 ^bc^	0.9 ± 0.0 ^cd^	0.6 ± 0.0 ^d^
C20:4n-3	0.7	0.8 ± 0.1	0.7 ± 0.0	0.7 ± 0.0	0.6 ± 0.0	0.5 ± 0.0
C20:5n-3 (EPA)	9.6	9.1 ± 0.1 ^a^	7.9 ± 0.2 ^ab^	6.4 ± 0.1 ^bc^	5.2 ± 0.1 ^cd^	3.7 ± 0.0 ^d^
C22:5n-3	1.9	2.2 ± 0.1	2.2 ± 0.0	2.2 ± 0.0	2.2 ± 0.1	2.1 ± 0.1
C22:6n-3 (DHA)	14.6	12.9 ± 0.1	13.2 ± 0.2	14.1 ± 0.1	13.2 ± 0.5	14.5 ± 0.4
Σn-3	30.1	27.7 ± 0.3	26.6 ± 0.2	26.1 ± 0.1	23.7 ± 0.5	23.2 ± 0.4
ΣPUFA	34.5	33.1 ± 0.3	32.9 ± 0.1	34.7 ± 0.1	34.4 ± 0.4	36.1 ± 0.3
DHA/EPA	1.5	1.4 ± 0.0	1.7 ± 0.1	2.2 ± 0.0	2.6 ± 0.1	3.9 ± 0.1
OA/DHA	1.3	1.3 ± 0.0	1.4 ± 0.0	1.5 ± 0.0	1.9 ± 0.1	1.8 ± 0.0
EPA/ARA	10.7	11.3 ± 0.1	8.8 ± 0.2	5.6 ± 0.4	4.1 ± 0.1	2.0 ± 0.1
n-6/n-3	0.2	0.2 ± 0.0	0.2 ± 0.0	0.3 ± 0.0	0.5 ± 0.0	0.6 ± 0.0 ^d^
PUFA/SFA	1.1	1.3 ± 0.0	1.3 ± 0.0	1.4 ± 0.0	1.4 ± 0.0	1.5 ± 0.0

Values are mean ± SD of three replicate samples. Means in row with different superscripts are significantly different (*p* < 0.05, Tukey’s test).

**Table 12:** Nutrient digestibility and productive value in greater amberjack fed experimental diets for 6 weeks.

Parameters (%)	C	TM25	TM50	TM75	TM100
Digestibility (%)					
Protein	92.3 ± 3.6	93.1 ± 2.8	92.5 ± 2.9	91.9 ± 2.2	91.7 ± 3.5
Fat	93.3 ± 2.6	94.7 ± 2.9	93.7 ± 3.1	92.1 ± 3.5	90.9 ± 5.9
Productive value (%)					
Protein	40.5 ± 2.9 ^a^	42.5 ± 0.1 ^a^	42.4 ± 2.1 ^a^	40.6 ± 2.3 ^a^	27.5 ± 3.9 ^b^
Fat	54.1 ± 10.4 ^a^	54.0 ± 5.2 ^a^	61.0 ± 2.6 ^a^	58.9 ± 4.9 ^a^	22.7 ± 5.7 ^b^
Phosphorus	33.4 ± 2.9 ^a^	39.8 ± 2.9 ^ab^	46.9 ± 2.8 ^abc^	57.9 ± 3.1 ^bc^	53.0 ± 13.8 ^c^

Abbreviations: control, C; TM25, TM50, TM75, and TM100 are diets replacing 25, 50, 75, and 100% of fish meal protein by tuna by-product meal. Values are mean ± SD of three replicate samples. Means in row with different superscripts are significantly different (*p* < 0.05, Tukey’s test).

**Table 13:** Phosphorus budget per unit body weight gain in greater amberjack fed with different diets for 6 weeks.

Parameters	C	TM25	TM50	TM75	TM100
Intake (g/kg WG)	16.9 ± 0.6 ^a^	15.4 ± 0.1 ^a^	13.1 ± 0.3 ^b^	10.7 ± 0.3 ^c^	11.1 ± 1.4 ^c^
Accumulation (g/kg WG)	5.6 ± 0.4	6.1 ± 0.5	6.2 ± 0.4	6.2 ± 0.3	5.7 ± 0.8
Loading (g/kg WG)	11.2 ± 0.7 ^a^	9.3 ± 0.2 ^b^	7.0 ± 0.4 ^c^	4.5 ± 0.4 ^d^	4.3 ± 1.1 ^d^

Abbreviations: control, C; TM25, TM50, TM75, and TM100 are diets replacing 25, 50, 75, and 100% of fish meal protein by tuna by-product meal. WG, weight gain. Values are mean ± SD of three replicate samples. Means in row with different superscripts are significantly different (*p* < 0.05, Tukey’s test).

**Table 14:** Plasma constituents in greater amberjack at end of 6-week rearing trial.

	C	TM25	TM50	TM75	TM100
GOT (U/L)	13.7 ± 3.5	9.3 ± 2.1	11.0 ± 3.6	9.0 ± 1.0	9.0 ± 1.4
GPT (U/L)	7.0 ± 0.0	6.0 ± 0.0	6.3 ± 0.6	6.7 ± 0.6	6.5 ± 0.7
TP (g/dL)	3.3 ± 0.1 ^a^	3.3 ± 0.3 ^a^	3.3 ± 0.2 ^a^	2.6 ± 0.0 ^b^	1.6 ± 0.4 ^c^
ALB (mg/dL)	0.9 ± 0.1 ^a^	0.9 ± 0.1 ^a^	0.9 ± 0.1 ^a^	0.6 ± 0.1 ^b^	0.3 ± 0.1 ^c^
TC (mg/dL)	210.0 ± 8.9 ^a^	301.3 ± 11.9 ^a^	197.0 ± 15.0 ^a^	134.3 ± 7.6 ^b^	74.0 ± 22.6 ^c^
TG (mg/dL)	28.3 ± 8.7 ^a^	51.7 ± 3.8 ^bc^	56.0 ± 7.5 ^c^	90.7 ± 10.0 ^d^	36.0 ± 7.1 ^ab^
GLU (mg/dL)	145.7 ± 19.4	134.3 ± 17.0	202.3 ± 62.6	181.0 ± 47.5	131.0 ± 5.7

Abbreviations: control, C; TM25, TM50, TM75, and TM100 are diets replacing 25, 50, 75, and 100% of fish meal protein by tuna by-product meal; GOT, glutamic oxaloacetic transaminase; GPT, glutamic pyruvic transaminase; TP, total protein; ALB, albumin; TC, total cholesterol; TG, triglyceride; GLU, glucose. Values are mean ± SD of nine samples. Means in row with different superscripts are significantly different (*p* < 0.05).

## Data Availability

The data will be made available from the corresponding author upon reasonable request.
